# Effect of platelet-rich fibrin gel combined with alginate dressing on healing of active venous leg ulcers: a randomized clinical trial

**DOI:** 10.3389/fmed.2026.1898776

**Published:** 2026-08-26

**Authors:** Feifei Cui, Rongting Wang, Zhen Xu, Qianqian Chen

**Affiliations:** 1 Department of Nursing, Wound Care Clinic, The Affiliated Dongyang People’s Hospital, Wenzhou Medical University, Dongyang, Zhejiang, China; 2 Department of Central Blood Bank, The Affiliated Dongyang People’s Hospital, Wenzhou Medical University, Dongyang, Zhejiang, China

**Keywords:** active venous leg ulcer, alginate dressing, ozonated oil, platelet-rich fibrin, wound healing

## Abstract

**Importance:**

Active venous leg ulcers (VLUs) represent a challenging chronic wound condition with prolonged healing trajectories, high recurrence rates, and substantial physical and psychological burden. Despite standard compression therapy, a significant proportion of VLUs fail to achieve timely closure, underscoring the need for effective conservative treatment options.

**Objective:**

To evaluate the efficacy and safety of autologous platelet-rich fibrin (PRF) gel combined with alginate dressing in promoting complete healing of active VLUs.

**Design, setting, and participants:**

A single-center, prospective, randomized controlled trial conducted between January 2023 and October 2025 at the vascular surgery and wound specialty outpatient clinics of a tertiary hospital in China. The 86 participants were patients with active VLUs (CEAP C6) who received standardized wound bed preparation and compression therapy. Participants were recruited through consecutive admissions.

**Interventions:**

Patients were randomly assigned 1:1 to receive either PRF gel combined with alginate dressing applied weekly (treatment group, *n* = 43) or ozonated oil combined with alginate dressing applied three times weekly (control group, *n* = 43) for 12 weeks.

**Main outcomes and measures:**

Primary efficacy endpoint was complete wound healing rate at 12 weeks. Secondary outcomes included healing efficacy (categorized as complete, markedly effective, effective, or ineffective), changes in pain intensity (Visual Analog Scale), and changes in anxiety levels (State–Trait Anxiety Inventory).

**Results:**

The PRF-alginate group achieved a significantly higher complete healing rate at 12 weeks compared with the control group (83.7% vs. 30.2%; risk difference, 53.5% [95% CI, 35.9–71.1%]; relative risk, 2.8 [95% CI, 1.7–4.4]). The median time to healing was 7.6 weeks (95% CI, 6.0–8.7) in the PRF group and was not reached in the control group (log-rank *p* = 0.006). PRF-alginate treatment was the strongest independent predictor of complete healing (odds ratio, 25.53 [95% CI, 4.95–131.50]), while baseline wound area was negatively associated with healing (odds ratio, 0.76 [95% CI, 0.64–0.90]). The PRF-alginate group showed a greater improvements in state anxiety scores (mean difference, 1.4 [95% CI, 0.0–2.8]) and trait anxiety scores (mean difference, 1.4 [95% CI, 0.1–2.7]) compared with the control group. No significant between-group difference was observed in pain scores (mean difference, 0.3 [95% CI, −0.1 to 0.7]). No serious adverse events were reported.

**Conclusions and relevance:**

The combination of platelet-rich fibrin gel and alginate dressing accelerates wound healing, shortens healing time, reduces pain and anxiety, and has a favorable safety profile in patients with active venous leg ulcers. This biologically grounded, technically simple, and cost-effective approach is a promising conservative treatment option for outpatient and primary care settings, particularly for elderly and geographically underserved populations who may not benefit adequately from conventional therapies requiring frequent clinic visits.

**Clinical trial registration:**

Clinicaltrials.gov Identifier: ChiCTR2400087844

## 1 Introduction

### 1.1 Venous leg ulcers: a global health challenge with inadequate solutions

Active venous leg ulcer (VLU), classified as C6 in the Clinical-Etiology-Anatomy-Pathophysiology (CEAP) system, represents the end-stage manifestation of chronic venous insufficiency (1). These full-thickness skin defects, typically located around the medial malleolus, fail to heal spontaneously and affect approximately 1–2% of the adult population in Western countries, with prevalence rising sharply with age (2, 3). Beyond their physical sequelae-severe pain, copious exudate, malodor, and recurrent infections VLUs inflict profound psychological morbidity. Patients frequently experience clinically significant anxiety, social isolation, and marked deterioration in quality of life (4, 5). The economic toll is equally alarming: prolonged healing trajectories, high recurrence rates, and repeated hospitalizations generate substantial healthcare expenditures, with annual costs exceeding billions of dollars in developed healthcare systems (2, 3, 6–8).

Despite decades of research and guideline directed care, the clinical outcomes of VLU management remain unsatisfactory. Compression therapy, the cornerstone of treatment, achieves complete healing in 60 to 80% of patients within 24 weeks, while approximately 20% of ulcers remain unhealed beyond 50 weeks (2, 9, 10). Alarmingly, more than half of patients may never attain complete closure with standard therapy alone (11, 12). When VLUs fail to reduce in area by 30–50% within 4 weeks of adequate treatment-a robust negative prognostic indicator, advanced interventions such as skin grafting, bioengineered substitutes, or negative pressure wound therapy are often considered (10, 13–17). However, these modalities are prohibitively expensive, require specialized infrastructure and expertise, and their superiority over optimized conservative care remains inconclusive, with reported efficacy rates below 60% within 12 weeks (10, 16, 18, 19). This therapeutic impasse is particularly consequential for the predominantly elderly VLU population, many of whom have limited mobility, multiple comorbidities, and face substantial barriers to accessing hospital based care (20). This slow and often incomplete healing is particularly challenging for elderly patients in remote areas, who face limited access to specialized wound care and frequent follow up (21, 22). Effective, safe, accessible, and cost-conscious conservative treatment strategies suitable for outpatient and primary care settings are urgently required (8). The search for fasteracting alternatives has thus led to the investigation of platelet rich concentrates, with platelet rich fibrin (PRF) emerging as a promising second generation preparation that overcomes many limitations of previous approaches (23, 24).

### 1.2 Platelet-rich fibrin: a biologically superior regenerative approach

Advances in regenerative medicine, particularly platelet concentrates, have catalyzed a paradigm shift in managing chronic, hard-to-heal wounds. Platelet-rich plasma (PRP), the first-generation platelet concentrate, has demonstrated efficacy in accelerating chronic wound healing through the delivery of supraphysiological concentrations of growth factors (24, 25). However, PRP therapy is constrained by several inherent limitations that have impeded its widespread clinical adoption (26–28). First, PRP preparation requires exogenous anticoagulants (typically bovine thrombin or calcium chloride), which introduce risks of immunological reactions, cross-infection, and may paradoxically delay normal wound healing processes. Second, the absence of standardized preparation protocols yields marked heterogeneity in platelet concentration, leukocyte content, and growth factor profiles across studies, contributing to conflicting efficacy data. Third, the high cost of commercial PRP kits and the requirement for repeated applications imposes substantial economic burden. Fourth, PRP administration necessitates repeated subcutaneous injections around the wound margins, causing considerable patient discomfort and limiting treatment acceptability, particularly in individuals with chronic painful ulcers.

Platelet-rich fibrin (PRF), a second-generation platelet concentrate developed by Choukroun and colleagues (28), fundamentally overcomes the limitations of first-generation platelet-rich plasma (29). Prepared via single-step centrifugation of autologous blood without any anticoagulants or exogenous additives, PRF eliminates immunogenic and cross-infection risks while substantially reducing costs (26, 30–32). The resulting gel possesses a unique three-dimensional fibrin architecture that mimics native extracellular matrix, serving dual functions: a biocompatible scaffold for cell adhesion, migration, and proliferation, and a sustained-release reservoir for endogenous growth factors, including platelet-derived growth factor (PDGF), transforming growth factor-*β* (TGF-β), vascular endothelial growth factor (VEGF), and epidermal growth factor (EGF), which are released continuously over 7–14 days (28, 31). The therapeutic efficacy of PRF in chronic wounds is underpinned by a multifaceted mechanism of action. First, its fibrin scaffold facilitates the recruitment and proliferation of fibroblasts, keratinocytes, and endothelial cells, essential for granulation tissue formation and re-epithelialization (32). Second, sustained growth factor release provides prolonged chemotactic and mitogenic stimulation: PDGF and TGF-*β* drive fibroblast recruitment and collagen synthesis, while VEGF promotes angiogenesis, counteracting the local ischemia characteristic of chronic venous ulcers (30). Third, the high concentration of viable leukocytes entrapped within the fibrin meshwork confers immunomodulatory and inherent antimicrobial properties, actively resolving the chronic, low-grade inflammation that perpetuates non-healing VLUs while providing local defense against colonization (19, 33).

Accumulating clinical evidence has confirmed that PRF significantly shortens healing time, improves granulation tissue quality, and reduces pain across diverse chronic wound etiologies, including diabetic foot ulcers, pressure injuries, burns, and refractory postsurgical wounds (30, 31, 34, 35). Systematic reviews and randomized trials further support its safety and efficacy in treating VLUs specifically (30, 36), establishing PRF as a clinically promising regenerative intervention.

### 1.3 Synergistic combination: PRF combined with alginate dressing

Despite compelling biological rationale, the clinical translation of PRF therapy for exudative VLUs remains hampered by inadequate moisture management. Conventional dressings-paraffin gauze covered with multiple dry layers-adhere to the wound bed, offer negligible absorption, and necessitate frequent changes, increasing nursing burden, patient discomfort, and costs. Poor exudate control leads to periwound maceration, extracellular matrix degradation, and delayed epithelialization. Alginate dressings, derived from natural seaweed, offer a clinically practical solution for wound management. They absorb wound exudate to form a gel-like matrix, which maintains a physiologically moist environment, reduces the risk of bacterial infection, supports autolytic debridement, and protects against periwound maceration (37).

The strategic combination of PRF with alginate dressing thus represents a biologically rational and pragmatically innovative approach. PRF delivers a sustained release “bioactive engine”-growth factors, immunomodulatory leukocytes, and a provisional fibrin matrix that actively drives angiogenesis, fibroblast proliferation, collagen synthesis, and re-epithelialization. Alginate provides the complementary “optimal environment”: superior exudate management, moisture balance, and infection protection. This synergy extends dressing change intervals to 7–10 days, reduces periwound complications and patient discomfort, and lowers treatment costs (31). These advantages are particularly critical for elderly, mobility- limited, and geographically isolated patients, for whom frequent clinic visits pose substantial barriers to care continuity.

### 1.4 Study rationale and clinical implications

This prospective randomized controlled trial evaluates the clinical efficacy and real-world applicability of autologous platelet-rich fibrin (PRF) gel combined with alginate dressing compared with conventional ozonated oil with alginate dressing in patients with active venous leg ulcers (VLUs). We hypothesized that early local intervention with this PRF-based regimen would achieve superior healing outcomes, including higher complete healing rates, shorter healing time, and greater improvements in patient-reported pain and anxiety, through three primary mechanisms: (1) resolution of chronic inflammation via PRF-derived immunomodulation, (2) enhanced angiogenesis and granulation tissue quality via sustained growth factor delivery, (3) accelerated re-epithelialization.

By integrating objective healing endpoints with validated patient-reported measures, this trial provides robust evidence for both the efficacy and practical implementation of PRF-alginate therapy in resource constrained outpatient and primary care settings. This biologically grounded, technically simple, and economical strategy can be readily adopted by community based providers, directly addressing the persistent therapeutic gap for elderly, mobility-limited, and geographically underserved patients with venous leg ulcers worldwide.

## 2 Methods

### 2.1 Study design

This was a prospective, single-center, randomized controlled trial conducted at the vascular surgery and wound specialty outpatient clinics of a tertiary hospital in China between January 2023 and October 2025. The study protocol was approved by the institutional ethics committee (Approval No.: 2023-YX-019) and was conducted in accordance with the Declaration of Helsinki and Good Clinical Practice guidelines. All enrolled participants provided written informed consent prior to study initiation. Study site personnel received standardized training on the protocol, PRF preparation technique, wound assessment procedures, and data collection methods at an investigator meeting, with training materials maintained and accessible throughout the study period. The trial consisted of a 12-week active treatment period, with outcomes assessed at baseline, weekly at the time of dressing changes, and at the 12-week endpoint.

### 2.2 Participants

Participants were recruited through consecutive admissions from the outpatient clinics. Recruitment materials included the following language: “Do you have a leg ulcer that has not healed for more than 4 weeks?” and “Are you looking for an effective treatment option that requires fewer clinic visits?” and “Would you like to participate in a clinical study evaluating a new regenerative therapy for chronic wounds?” Enrollment ended when recruitment goals were achieved.

Eligible participants were adults aged 18 years or older with active venous leg ulcers (CEAP C6 classification) confirmed by clinical examination or ultrasound-documented venous insufficiency. Additional inclusion criteria were: ulcer duration ≥4 weeks; ulcer size between 4–30 cm^2^; clean wound bed after debridement without severe infection; ankle-brachial index ≥0.8; ability to tolerate compression therapy; and provision of informed consent. Exclusion criteria included platelet count <100 × 10^9^/L, long-term anticoagulant use, severe peripheral arterial disease, significant organ dysfunction, immunosuppressant use, or acute cellulitis. Participants could be withdrawn from the study for the following reasons: transfer to another hospital, or voluntary withdrawal by the patient or family members. To minimize participation barriers and promote protocol adherence throughout the 12-week study period, all study related interventions-including PRF preparation materials and ozonated oil dressings were provided to participants at no cost. This facilitated consistent treatment delivery and contributed to the high retention rate observed in both study groups.

### 2.3 Sample size

The sample size was calculated using PASS 15 Power Analysis and Sample Size software *(NCSS, LLC, Kaysville, UT, United States,* ncss.com/software/pass) based on a two-group superiority test of proportions. The calculation assumed a complete healing rate of 78.57% in the experimental group and 41.67% in the control group, derived from a prior pilot study (data not shown). With a one-sided significance level of *α* = 0.05, power (1-*β*) of 90%, and a 1:1 allocation ratio, the required sample size was 34 participants per group. Accounting for a 20% dropout rate, the final target enrollment was 43 participants per group, for a total of 86 participants.

### 2.4 Study procedures

Eligible participants were recruited and screened by vascular surgeons according to predefined inclusion and exclusion criteria, and were sequentially numbered in order of admission. A computer generated random allocation sequence using block randomization was created by an independent statistician using SPSS software *(version 26.0, IBM Corp, Armonk, NY, United States)*, assigning participants in a 1:1 ratio to either the PRF-alginate group or the control group. Participant identification numbers and corresponding group assignments were sealed in sequentially numbered, opaque, brown envelopes. After completion of baseline assessments, group allocation was performed by a research nurse who was not involved in treatment or outcome assessment. Due to inherent differences in the physical appearance and application frequency between PRF gel and ozonated oil, blinding of participants and treating clinicians was not feasible. However, outcome assessors responsible for wound area measurements from standardized digital photographs and data analysts were kept blinded to group assignment throughout the study period.

### 2.5 Interventions

All wounds were managed based on evidence-based practice, moist wound healing principles, and the TIME framework. Necrotic tissue (e.g., slough) was removed, and infection was controlled until fresh granulation tissue appeared (wound bed preparation). Patients followed vascular surgeons’ advice regarding venous function medications and compression therapy. Baseline data on venous function, pain, and anxiety were collected. Researchers assessed treatment weekly, recording pain, wound area, granulation tissue, and epithelialization. Digital photos were taken at enrollment and each dressing change. Ulcer length, width, and depth were measured using the “clock face” method. Treatment continued for 12 weeks. If healed before or at week 12, the healing date was recorded. Patients unhealed at week 12 continued treatment but were excluded from final analysis. Patients received instruction via animated videos/pictures on proper use of self-adhesive elastic bandages, skin ointments (moisturizing/anti-itch), leg elevation, and calf muscle pump exercises.

#### 2.5.1 Control group

Following wound bed preparation, medical-grade ozonated oil *(BAODI®; Shaanxi Hailin Zhengde Biomedical Co., Ltd., Xi’an, Shaanxi, China)* was applied evenly at a dose of 1 mL per cm^2^ of wound area. For moderately to heavily exuding wounds, an ozonated oil-impregnated alginate dressing was applied. For minimally exuding wounds, Vaseline gauze was used as the secondary layer, secured with a compression bandage. Dressings were changed three times per week for 12 weeks.

#### 2.5.2 Treatment group

After wound bed preparation, PRF gel was prepared according to wound size. Based on wound dimensions, 5 mL of venous blood was drawn from the median cubital vein into a vacuum tube without anticoagulant. Each 5 mL tube yielded a cylindrical PRF gel approximately 0.8 cm in diameter and 2.0 cm in height. Blood volume was estimated pre-draw based on wound volume/area. Within 60–90 s after blood draw, centrifugation was performed at a radius of 15 cm, 2,700–3,000 rpm for 10–12 min, followed by 10 min of rest. Three layers became visible: upper yellowish platelet-poor plasma, bottom red cell debris, and middle yellowish PRF gel. After removing the tube cap, PRF gel and some red cell debris were aspirated using a syringe needle (negative pressure suction). Most of the underlying red cell layer was absorbed with sterile gauze, leaving about 1.0 mm of red cell debris at the interface to avoid loss of growth factors and leukocytes A schematic illustration of the key steps involved in PRF therapy for venous leg ulcers is provided in Figure 1.

**Figure 1:**
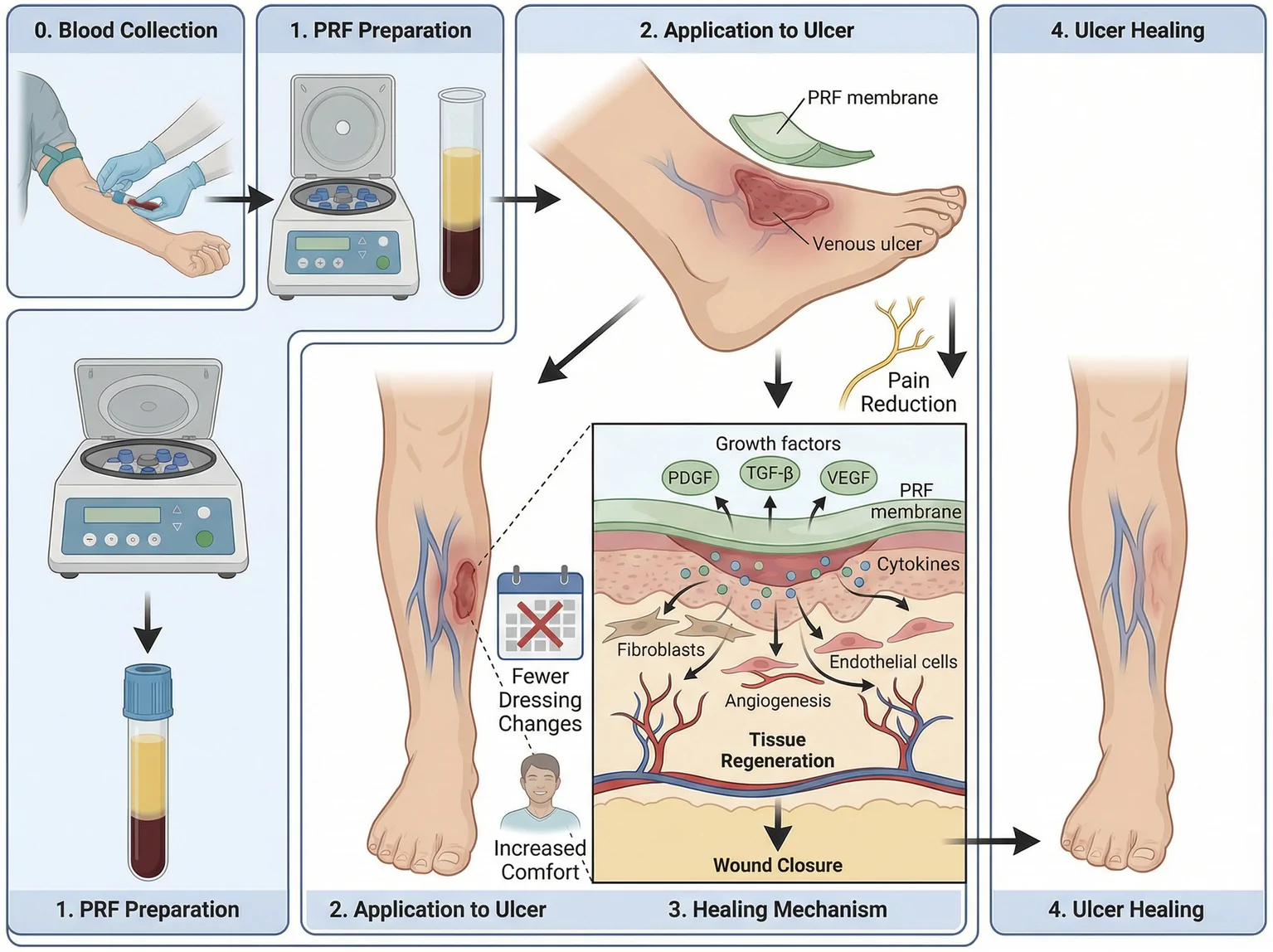
Preparation, application, and proposed mechanism of action of Platelet-Rich Fibrin (PRF) in venous leg ulcer healing. Schematic illustration of the key steps involved in PRF therapy for venous leg ulcers. (0) Blood collection from the patient. (1) Preparation of PRF membrane after centrifugation. (2) Application of the PRF membrane to the venous ulcer surface, which reduces pain and increases patient comfort compared with conventional dressings. (3) Proposed biological mechanisms: PRF releases growth factors (PDGF, TGF-β, VEGF) and cytokines, which act on fibroblasts and endothelial cells, leading to enhanced angiogenesis, tissue regeneration, and ultimately wound closure. Fewer dressing changes and improved comfort are additional clinical benefits.

The volume of PRF gel obtained from each 5-mL blood collection was approximately 1.0–1.5 mL after centrifugation and separation. The total volume of PRF gel applied to the wound was calculated based on wound area, with a target application density of approximately 0.2–0.3 mL of PRF gel per cm^2^ of wound surface. For wounds with irregular shapes or deep cavities, the gel was carefully molded to achieve uniform contact with the entire wound bed. In this study, the total PRF volume applied per session ranged from 2.0 to 5.0 mL, depending on individual wound dimensions. The PRF gel was prepared fresh at each treatment visit and applied within 30 min of preparation to ensure optimal bioactivity. No exogenous additives (e.g., thrombin, calcium chloride, or anticoagulants) were used during preparation.

The prepared PRF gel was applied directly to the wound bed at a thickness of approximately 2–3 mm and covered with an alginate dressing pre-moistened with 1–2 mL of the patient’s own platelet-poor plasma (the supernatant from PRF preparation). The alginate dressing used in this study was Biatain® Alginate (manufactured by Advanced Medical Solutions Ltd., Winsford, Cheshire, United Kingdom; distributed by Coloplast (China) Medical Supplies Co., Ltd., Beijing, China). It is a medical-grade, sterile, non-woven pad composed of calcium alginate and carboxymethylcellulose (CMC), derived from brown seaweed. The dressing is designed to absorb wound exudate and create a moist wound healing environment. The specific model used was 3,710 (10 cm × 10 cm), which was trimmed to fit the wound dimensions as needed, typically extending 1–2 cm beyond the wound margins to ensure full coverage of the wound bed and periwound area. All dressings were used within their expiration date and stored according to the manufacturer’s instructions (15 °C–25 °C, protected from sunlight). The product is registered under Chinese Medical Device Registration No. 20163145062. The dressing was changed weekly, and the volume of PRF was re-calculated at each visit based on the current wound area, ensuring that the PRF dose was consistently adjusted to the changing wound dimensions. The secondary layer was sterile gauze, secured with a compression bandage. An image of the PRF-alginate combination dressing, showing its layered structure and appearance prior to application, is provided as Supplementary Figure 1.

### 2.6 Study end points

The primary outcome was the complete wound healing rate at the treatment phase, defined as 100% epithelialization.

Secondary outcomes included: (1) healing efficacy categorized as complete healing (100% epithelialization), markedly effective (≥80% area reduction), effective (≥40 to <80% area reduction), or ineffective (<40% area reduction); (2) total effective rate (complete + markedly effective); (3) wound healing rate [(baseline area - final area)/baseline area × 100%]; (4) anxiety levels assessed by the State–Trait Anxiety Inventory (STAI); (5) pain intensity measured by Visual Analog Scale (VAS, 0–10); and (6) adverse events including skin allergy, infection, or wound deterioration.

Wound area was measured weekly from standardized digital photographs using the clock-face method. Pain and Anxiety scores were assessed at baseline and 12-weeks (or at the time of healing if this occurred earlier). Safety monitoring continued throughout the study period.

### 2.7 Statistics

The primary efficacy analysis was performed on the intention-to-treat population, including all randomized participants. The complete healing rate at 12 weeks was compared between groups using the χ^2^ test. Risk differences (RDs) and 95% confidence intervals (CIs) were calculated to quantify the absolute treatment effect. For continuous secondary outcomes (anxiety scores, pain scores), between-group comparisons were performed using independent-samples t tests or Mann–Whitney *U* tests based on distribution normality. Within-group changes from baseline to week 12 (or healing date) were assessed using paired t tests or Wilcoxon signed-rank tests. A binary logistic regression model was constructed to identify independent predictors of complete healing, with treatment group as the primary explanatory variable. The model included the following covariates based on clinical relevance and baseline imbalances: ulcer etiology, comorbidities, venous clinical severity score, and initial ulcer area. In accordance with the prespecified statistical analysis plan, the main inferences were based on this model regardless of the significance of any potential interaction effects. Adjusted odds ratios (ORs) and 95% CIs were reported. All statistical tests were 2-sided, with *p* < 0.05 considered statistically significant. Data processing and analysis were performed using R version 4.3.3 (2024-02-29), along with Zstats 2.0^1^.

## 3 Results

### 3.1 Enrollment and follow-up

A total of 135 patients were assessed for eligibility. Of these, 86 (63.7%) were randomly assigned to receive PRF (*n* = 43) or control (*n* = 43). In the PRF group, 36 patients (83.7%) completed the trial (defined as either complete healing before week 12 or completion of the 12-week follow-up), compared with 15 patients (34.9%) in the control group (Figure 2). Baseline demographic and wound characteristics were similar between groups (Table 1). All randomized participants received the allocated intervention and were included in the intention-to-treat analysis.

**Figure 2:**
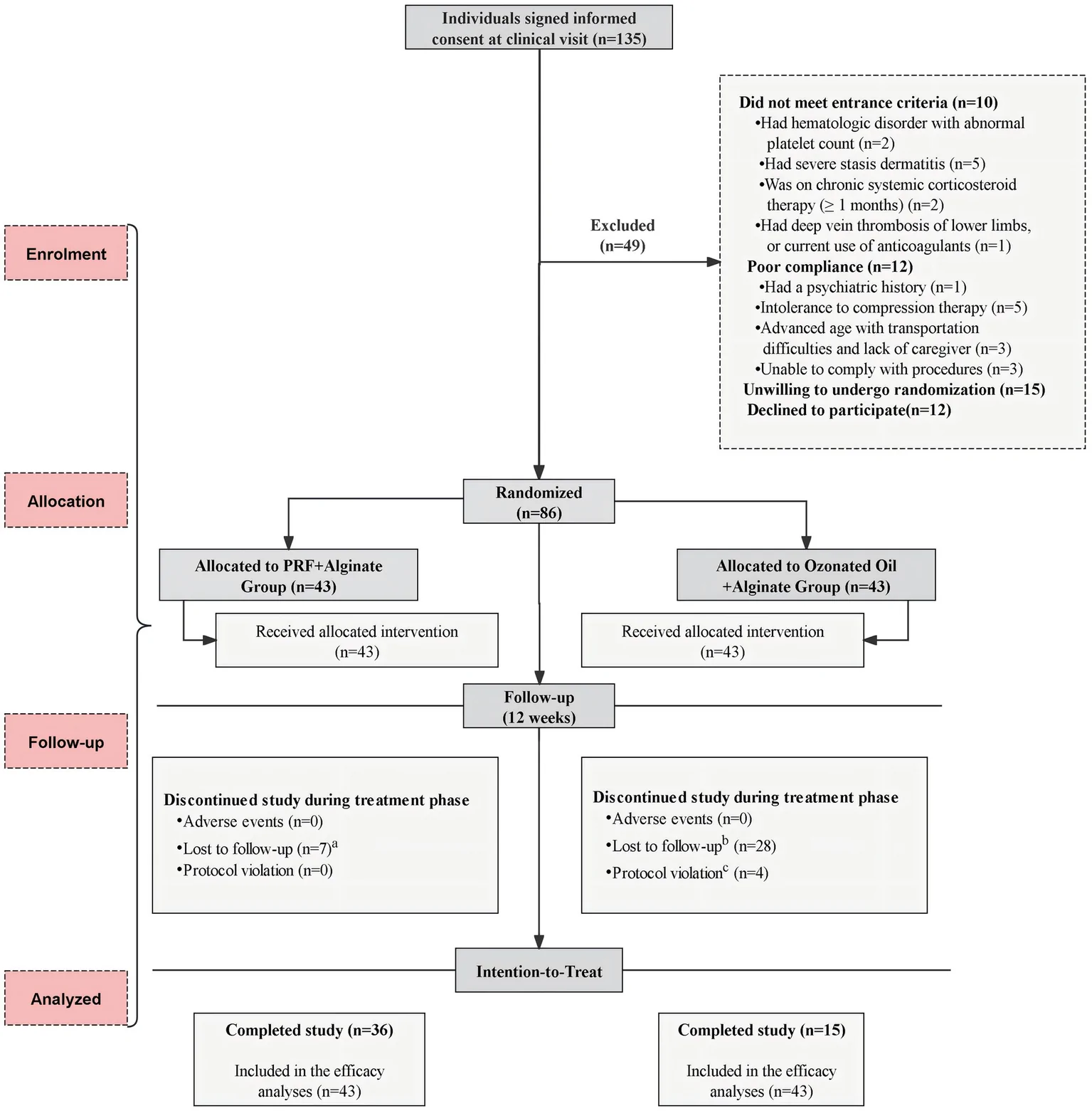
PRF vs control for venous leg ulcer healing trial flow diagram. ^a^Seven patients discontinued hospital visits during the treatment phase. Reasons included transportation difficulties, lack of family accompaniment, and substantial wound improvement leading to transfer to local community hospitals for routine maintenance care. All patients received telephone counseling on post-treatment wound care. ^b^Twenty-eight patients discontinued (*n* = 24 for similar reasons; *n* = 4 for lack of improvement at 4 weeks). All received telephone wound care counseling. ^c^Four patients in the control group with lack of noticeable improvement at 4 weeks were switched to PRF treatment but remained in the control group for analysis, with outcomes assessed according to their last wound healing status.

**Table 1:** Participant characteristics.

Variables	Total (*n* = 86)	Control group (*n* = 43)	PRF group (*n* = 43)	Statistic	*p*-value
Age, mean (SD), y	64.07 (12.20)	63.70 (10.39)	64.44 (13.89)	*t* = −0.28	0.779
Gender, *n* (%)				χ^2^ = 1.84	0.175
Women	30 (34.88)	18 (41.86)	12 (27.91)		
Men	56 (65.12)	25 (58.14)	31 (72.09)		
Location, *n* (%)				χ^2^ = 0.50	0.777
Ankle	33 (38.37)	15 (34.88)	18 (41.86)		
Pretibial	39 (45.35)	21 (48.84)	18 (41.86)		
Gaiter area	14 (16.28)	7 (16.28)	7 (16.28)		
Causes of Wound Formation^a^, *n* (%)				χ^2^ = 3.28	0.070
Venous	56 (65.12)	24 (55.81)	32 (74.42)		
Mixed	30 (34.88)	19 (44.19)	11 (25.58)		
Comorbidities, *n* (%)				χ^2^ = 7.58	0.056
None	37 (43.02)	24 (55.81)	13 (30.23)		
Hypertension	16 (18.60)	7 (16.28)	9 (20.93)		
Diabetes	17 (19.77)	8 (18.60)	9 (20.93)		
Other^b^	16 (18.60)	4 (9.30)	12 (27.91)		
Timetime of duration, median (IQR), wk	6.57 (4.43, 10.86)	6.14 (4.43, 9.36)	8.86 (4.50, 13.07)	*Z* = −1.80	0.072
Vein score, mean (SD)	13.48 (2.15)	13.05 (2.08)	13.91 (2.16)	*t* = −1.88	0.063
Pre-area, mean (SD)	9.56 (4.41)	9.18 (3.99)	9.94 (4.81)	*t* = −0.80	0.427
Pre-VAS sorce, mean (SD)	3.17 (0.64)	3.14 (0.68)	3.21 (0.60)	*t* = −0.51	0.614
PreS-AI Score, mean (SD)	51.70 (3.37)	52.14 (3.38)	51.26 (3.30)	*t* = 1.22	0.226
PreT-AI Score, mean (SD)	53.70 (3.37)	54.23 (3.36)	53.26 (3.33)	*t* = 1.26	0.208

a Venous causes were primarily attributed to chronic venous insufficiency, with subsequent friction and scratching leading to infection and improper wound management, resulting in wound expansion and delayed healing. Mixed causes referred to wounds that developed on the basis of chronic venous insufficiency, where trauma led to wound infection and improper management, resulting in wound expansion and delayed healing.

b Comorbidities were categorized as follows: (1) hypertension alone; (2) diabetes alone; and (3) other conditions, including mixed diagnoses (eg, coronary heart disease, post-stroke sequelae) or hypertension and/or diabetes combined with additional diseases.

IQR, interquartile range. wk, week.

During the treatment phase, premature discontinuation of hospital visits before complete healing occurred in 7 patients (16.3%) in the PRF group and 28 patients (65.1%) in the control group. Among control group participants, 13 achieved complete healing, and 2 completed the full 12-week treatment without complete healing but with a final wound area reduction >80%. Patients who switched to PRF therapy due to poor response (*n* = 4) remained in their original assigned group, with their last assessment carried forward. The primary reasons for discontinuation were transportation difficulties and marked wound improvement, with patients opting for continued care at local community hospitals. Detailed stratification of wound area reduction at the time of discontinuation is provided in Supplementary Table 1. All patients who discontinued hospital visits received telephone counseling on post-treatment wound care.

### 3.2 Wound healing outcomes

#### 3.2.1 Complete healing

The PRF group demonstrated significantly higher complete healing rates at the primary endpoint compared with the control group (83.7% [36/43] vs. 30.2% [13/43]; risk difference [RD], 53.5% [95% CI, 35.9–71.1%]; relative risk [RR], 2.8 [95% CI, 1.7–4.4], number needed to treat [NNT] = 2 (Table 2).

**Table 2:** Primary and secondary outcomes.

Outcome	Control Group (*n* = 43)	PRF Group (*n* = 43)	Risk Difference, % (95% CI)	Relative Risk (95% CI)
Primary outcome				
Complete healing, *n* (%)^a^	13 (30.2)	36 (83.7)	53.5 (35.9–71.1)	2.8 (1.7–4.4)
Treatment response rate, *n* (%)^b^	25 (58.1)	38 (88.4)	30.3 (12.6–47.9)	1.5 (1.2–2.0)
Secondary outcomes				
Change in VAS, mean (SD)^c^	−1.8 (1.1)	−2.1 (1.0)	0.3 (−0.1–0.7)	
Change in S-AI, mean (SD)	−8.4 (3.5)	−9.8 (3.2)	1.4 (0.0–2.8)	
Change in T-AI, mean (SD)	−8.1 (3.4)	−9.5 (3.1)	1.4 (0.1–2.7)	

^a^Intention-to-treat analysis: loss to follow-up considered as not healed. ^b^“Complete healing” and “marked improvement” were combined as “treatment response” for analysis. ^c^Change = Post - Pre. Negative value indicates improvement.

#### 3.2.2 Wound area reduction

The PRF group demonstrated a significantly greater reduction in wound area compared with the control group. The mean percentage reduction in wound area was 91.2% (SD, 18.3%) in the PRF group versus 58.4% (SD, 32.1%) in the control group (mean difference (MD), 32.8% [95% CI, 21.5–44.1%].

The proportion of patients achieving clinically meaningful wound area reduction thresholds was significantly higher in the PRF group. The proportion achieving ≥50% wound area reduction was 95.3% (41/43) in the PRF group compared with 60.5% (26/43) in the control group (RD, 34.8% [95% CI, 19.9–49.7%]; The proportion achieving ≥75% wound area reduction was 86.0% (37/43) in the PRF group versus 41.9% (18/43) in the control group (RD, 44.1% [95% CI, 26.7–61.5%].

### 3.3 Treatment response rates

When considering clinical improvement (defined as complete healing or marked improvement, i.e., ≥80% area reduction), the PRF group achieved a significantly higher rate than the control group (88.4% vs. 58.1%; RD, 30.3% [95% CI, 12.6–47.9%]; RR, 1.5 [95% CI, 1.2–2.0]) (Table 2). Conversely, the control group had higher proportions of patients with only mild improvement (34.9% [15/43] vs. 11.6% [5/43]) and no improvement (7.0% [3/43] vs. 0% [0/43]) compared with the PRF group (Figure 3).

**Figure 3:**
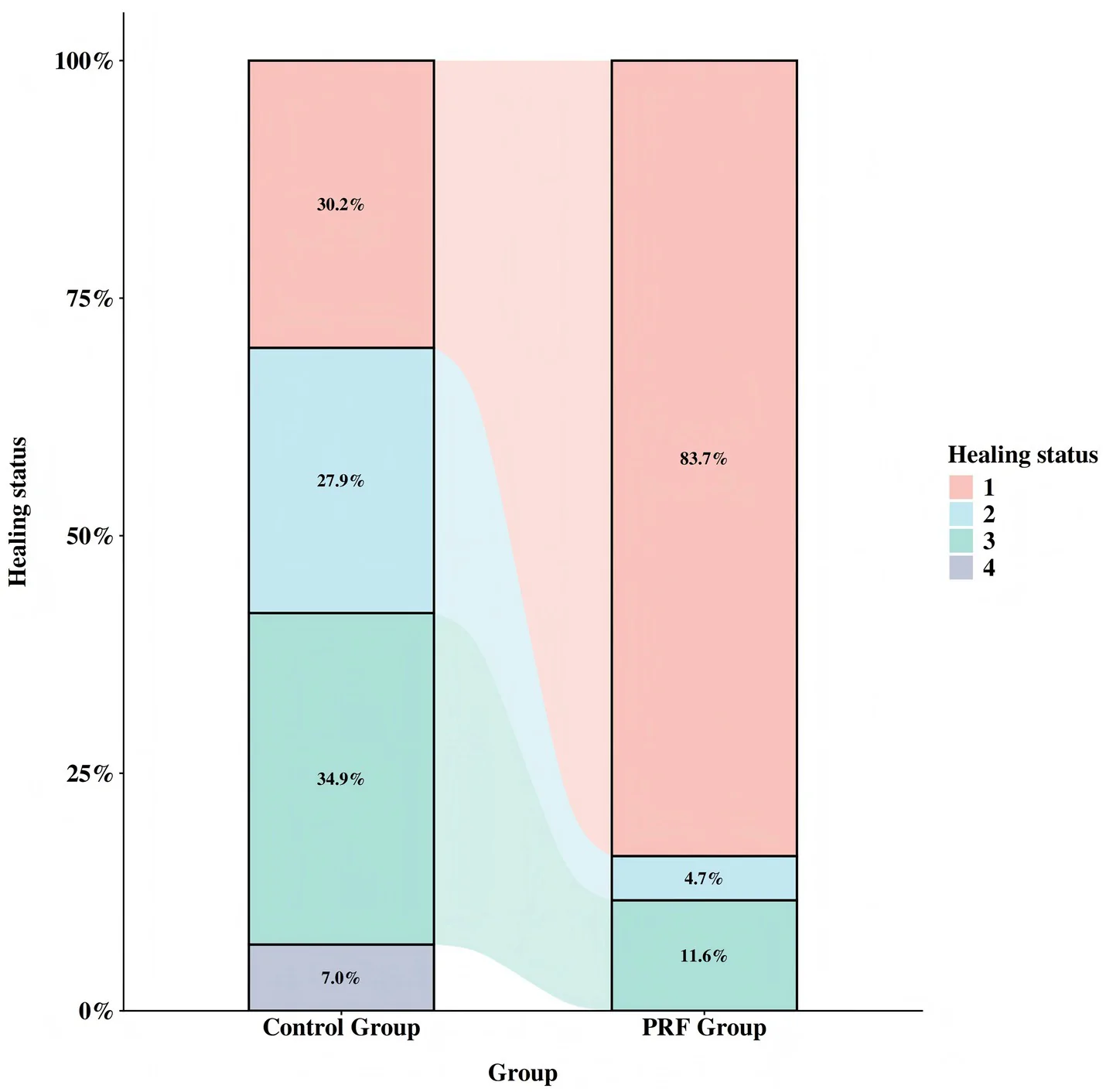
Distribution of wound healing status. Stacked bar chart showing the proportion of patients in each healing status category at final follow-up. Healing status was defined as follows: 1 = Complete healing (wound fully epithelialized, no exudate); 2 = Marked improvement (≥80% reduction in wound area); 3 = Improvement (≥40 to <80% reduction in wound area); 4 = No improvement (<40% reduction).

### 3.4 Time to wound healing

Kaplan–Meier survival analysis was performed to compare the time to complete wound healing between the two treatment groups. In the PRF group, the median time to complete healing was 7.6 weeks (95% CI, 6.0–8.7), with 36 of 43 participants (83.7%) achieving complete closure within the follow-up period. In the control group, the median time to healing could not be estimated because fewer than 50% of participants achieved complete healing during follow-up (13 of 43 participants [30.2%] achieved closure). Kaplan–Meier analysis revealed significantly faster wound healing in the PRF group compared with the control group (log-rank *p* = 0.006), the hazard ratio for complete healing with PRF-alginate therapy versus control was 2.33 (95% CI, 1.23–4.40). The Kaplan–Meier curves for time to complete healing are presented in Figure 4.

**Figure 4:**
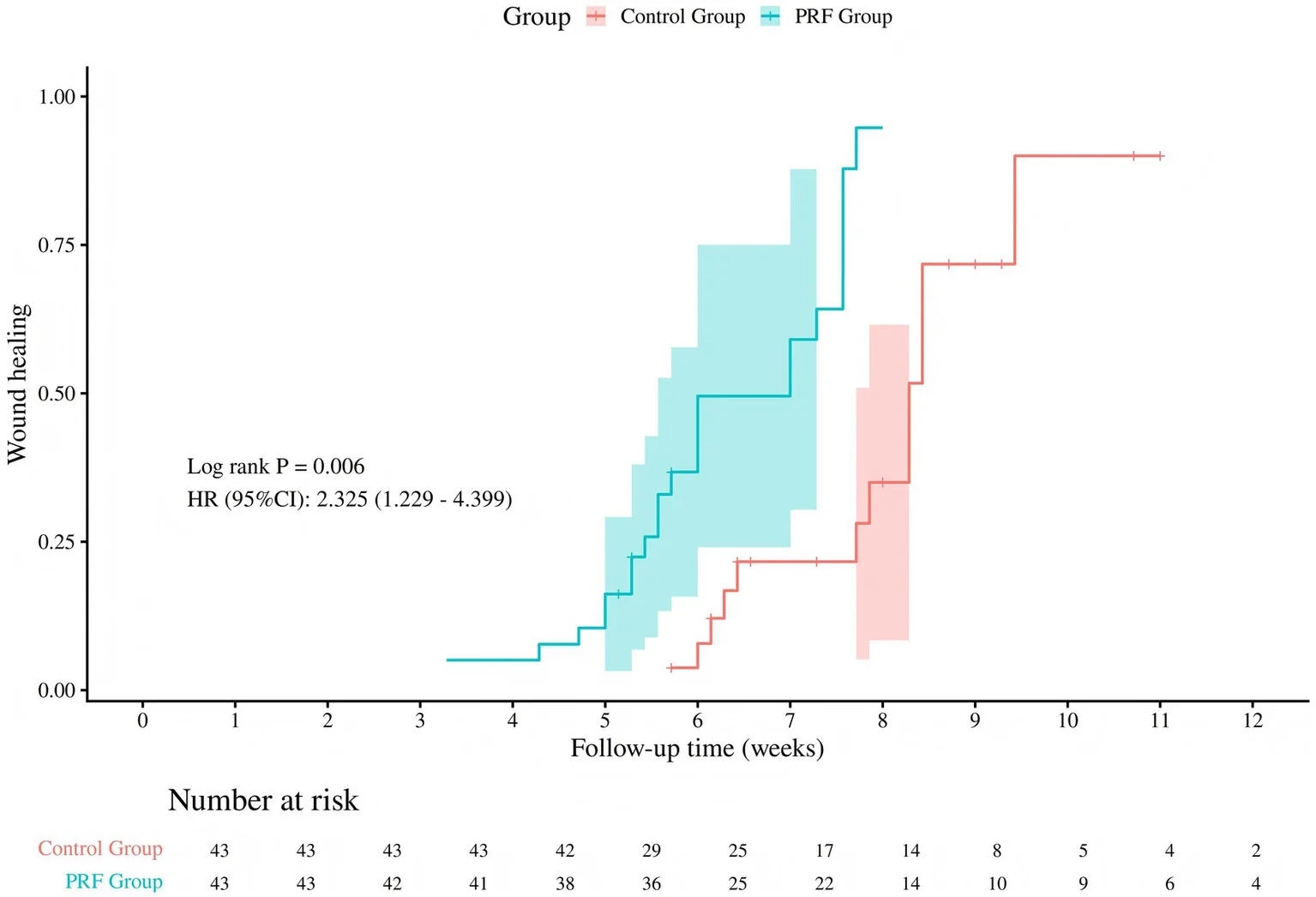
Kaplan–Meier curves for time to complete wound healing. Shown are the cumulative probabilities of complete wound healing over 12 weeks of follow-up for the PRF group (blue) and the control group (red). The PRF group demonstrated a significantly shorter time to healing compared with the control group (log-rank *p* = 0.006, hazard ratio [HR] = 2.325). The number of patients at risk at each time point is provided below the x-axis.

### 3.5 Secondary outcomes

#### 3.5.1 VAS reduction

The mean reduction in VAS score was comparable between the two groups, with no significant difference observed. The PRF group achieved a mean VAS reduction of 2.1 (SD, 1.0) compared with 1.8 (SD, 1.1) in the control group (MD, 0.3 [95% CI, −0.1 to 0.7]; *p* = 0.12). The proportion of patients achieving a clinically meaningful VAS reduction (defined as ≥2 points) was 62.8% (27/43) in the PRF group versus 51.2% (22/43) in the control group (RD, −11.6% [95% CI, −32.2 to 9.0%]; *p* = 0.27), Table 2.

### 3.6 S-AI and T-AI score reduction

The PRF group showed a greater reduction in S-AI scores compared with the control group, although this difference did not reach statistical significance. The mean S-AI reduction was 9.8 (SD, 3.2) in the PRF group versus 8.4 (SD, 3.5) in the control group (MD, 1.4 [95% CI, 0.0 to 2.8]; *p* = 0.05). The proportion of patients achieving a reduction of ≥10 points in S-AI score was 62.8% (27/43) in the PRF group compared with 48.8% (21/43) in the control group (RD, 14.0% [95% CI, −6.7 to 34.7%]; *p* = 0.19).

The PRF group demonstrated a significantly greater reduction in T-AI scores compared with the control group. The mean T-AI reduction was 9.5 (SD, 3.1) in the PRF group versus 8.1 (SD, 3.4) in the control group (mean difference, 1.4 [95% CI, 0.1 to 2.7]; *p* = 0.04). The proportion of patients achieving a reduction of ≥10 points in T-AI score was 67.4% (29/43) in the PRF group compared with 53.5% (23/43) in the control group (RD, 13.9% [95% CI, −6.2 to 34.0%]; *p* = 0.18), Table 2.

### 3.7 Factors associated with complete wound healing

Univariate and multivariate logistic regression analyses were performed to identify factors independently associated with complete wound healing (Table 3). In the multivariate model, PRF-alginate treatment was the strongest independent predictor of complete healing (OR, 25.53; 95% CI, 4.95–131.50; *p* < 0.001), with the control group as the reference. Baseline wound area was also independently associated with healing (OR, 0.76; 95% CI, 0.64–0.90; *p* = 0.002). No other variables, including gender, wound location, etiology, comorbidities, age, wound duration, or venous severity score, achieved statistical significance in the multivariate model (all *p* > 0.05).

**Table 3:** Univariate and multivariate logistic regression analysis of factors associated with complete wound healing.

Variables	*β*	S.E	*Z*	*p*	OR (95% CI)	*β*	S.E	Z	*P*	OR (95% CI)
Group										
Control Group					1.00 (Reference)					1.00 (Reference)
PRF Group	2.31	0.51	4.50	<0.001	10.10 (3.69 ~ 27.63)	3.24	0.84	3.87	<0.001	25.53 (4.95 ~ 131.50)
Gender										
Women					1.00 (Reference)					1.00 (Reference)
Men	0.57	0.46	1.24	0.213	1.77 (0.72 ~ 4.33)	1.44	0.75	1.94	0.053	4.23 (0.98 ~ 18.24)
Location										
Ankle					1.00 (Reference)					1.00 (Reference)
Pretibial	0.30	0.48	0.63	0.526	1.35 (0.53 ~ 3.44)	0.92	0.73	1.26	0.208	2.51 (0.60 ~ 10.48)
Gaiter area	0.23	0.64	0.35	0.724	1.25 (0.36 ~ 4.42)	1.30	0.98	1.33	0.185	3.66 (0.54 ~ 24.95)
Causes of wound formation										
Venous					1.00 (Reference)					1.00 (Reference)
Mixed	−0.15	0.45	−0.34	0.735	0.86 (0.35 ~ 2.09)	0.14	0.71	0.20	0.841	1.15 (0.29 ~ 4.66)
Comorbidities										
None					1.00 (Reference)					1.00 (Reference)
Hypertension	1.28	0.64	2.02	0.044	3.61 (1.04 ~ 12.60)	1.62	0.86	1.88	0.060	5.07 (0.94 ~ 27.45)
Diabetes	0.85	0.60	1.43	0.154	2.35 (0.73 ~ 7.58)	1.50	0.86	1.74	0.081	4.47 (0.83 ~ 24.07)
Other	1.96	0.72	2.71	0.007	7.12 (1.72 ~ 29.46)	2.19	1.15	1.91	0.057	8.90 (0.94 ~ 84.13)
Age	−0.02	0.02	−1.26	0.206	0.98 (0.94 ~ 1.01)	−0.02	0.03	−0.63	0.529	0.98 (0.92 ~ 1.04)
Timetime of duration (week)	0.03	0.04	0.83	0.407	1.04 (0.95 ~ 1.13)	−0.09	0.07	−1.25	0.212	0.92 (0.80 ~ 1.05)
Vein score	0.08	0.10	0.82	0.411	1.09 (0.89 ~ 1.33)	0.06	0.16	0.38	0.703	1.06 (0.77 ~ 1.47)
Pre-area	−0.11	0.05	−2.17	0.030	0.89 (0.80 ~ 0.99)	−0.28	0.09	−3.11	0.002	0.76 (0.64 ~ 0.90)

### 3.8 Safety

Safety monitoring was performed throughout the treatment period. In the control group, 2 of 43 participants (4.7%) developed mild periwound papular rash accompanied by tolerable pruritus, which resolved with topical corticosteroid application. Five participants (11.6%) reported transient local stinging sensation that resolved within 24 h after dressing change, and 3 participants (7.0%) experienced mild periwound maceration. In the PRF group, 5 participants (11.6%) developed mild periwound maceration, and 3 participants (7.0%) developed mild periwound papular rash with tolerable pruritus that resolved following topical corticosteroid therapy. Additionally, 2 participants (4.7%) in the PRF group developed granulation tissue edema, which was managed with local treatment and did not affect subsequent wound healing; no such events were observed in the control group. No serious adverse events, including severe allergic reactions, wound infection exacerbation, or ulcer deterioration, were reported in either treatment group. No participant discontinued the study due to adverse events (Table 4).

**Table 4:** Adverse events during the treatment period.

Adverse event	Control group (*n* = 43)	PRF-alginate group (*n* = 43)	Risk difference (95% CI)	*p*-value
Any adverse event	10 (23.3)	10 (23.3)		
Local skin reactions				
Mild periwound papular rash	2 (4.7)	3 (7.0)	−2.3% (−12.4 to 7.8%)	1.000
Mild periwound maceration	3 (7.0)	5 (11.6)	−4.7% (−17.3 to 7.9%)	0.72
Treatment-related sensations				
Transient local stinging sensation^a^	5 (11.6)	0	11.6% (2.2 to 21.0%)	0.06
Wound healing-related				
Granulation tissue edema	0	2 (4.7)	−4.7% (−13.0 to 3.6%)	0.50
Serious adverse events	0	0		
Severe allergic reactions	0	0		
Wound infection exacerbation	0	0		
Ulcer deterioration	0	0		

^a^Wound bed preparation, was performed in both treatment groups according to standard clinical protocols. Such procedures inherently carry a risk of mild procedural pain, which is unrelated to the study interventions. The adverse event “transient local stinging sensation” was specifically recorded when participants reported pain or burning sensations that occurred during or immediately after the application of study dressings. All reported stinging sensations were mild in intensity, resolved spontaneously within 24 h following dressing change, and did not necessitate intervention or study discontinuation.

Data are presented as *n* (%). *p* values were calculated using Fisher exact test.

### 3.9 Case illustrations

To further illustrate the clinical course and healing trajectory associated with PRF therapy, representative case examples are provided.

#### 3.9.1 Case 1

A 78-year-old man with a history of diabetes and venous sclerosis presented with an active venous ulcer located on the right pretibial region. The ulcer had been present for more than 6 months. The patient declined hospitalization for skin grafting and preferred conservative treatment.

At initial evaluation, the wound bed consisted of 75% fibrotic slough and 25% granulation tissue, with dimensions of 7.4 cm × 4.0 cm (area, 29.6 cm^2^). Conservative sharp debridement was performed to remove non-viable tissue until granulation tissue exceeded 90%. After 4 weeks of wound bed preparation, the patient provided informed consent for PRF therapy, which was provided free of charge. At the start of PRF treatment, the wound area was 27.75 cm^2^ (7.5 cm × 3.7 cm). After 2 weeks of weekly PRF applications, the area decreased to 18.0 cm^2^ (35.1% reduction). After 4 weeks, the area was 6.9 cm^2^ (75.1% reduction). A total of 6 PRF applications (one per week) were administered until complete wound closure was achieved. Representative clinical photographs are shown in Figure 5.

**Figure 5:**
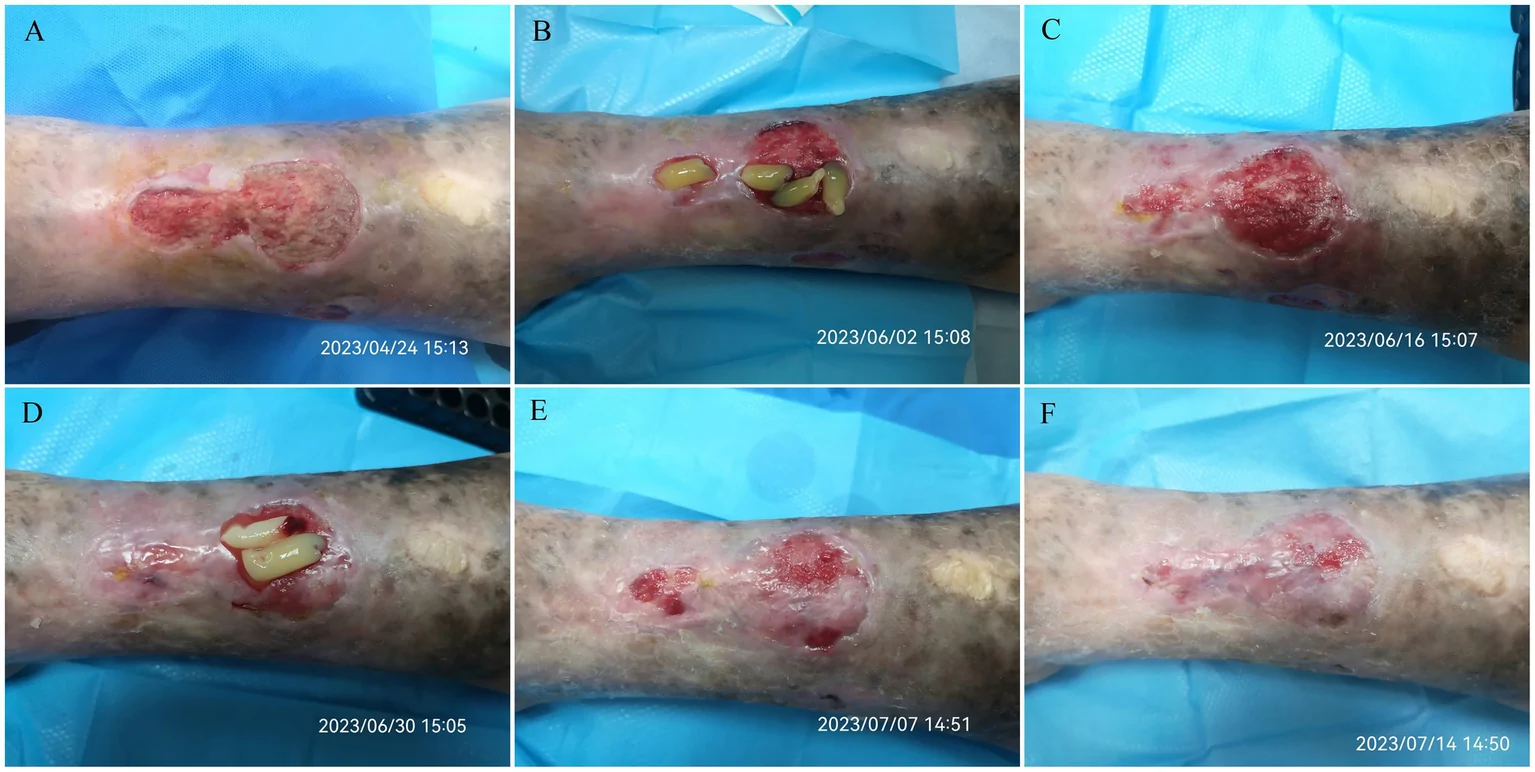
Clinical photographs of a venous leg ulcer treated with PRF-alginate therapy (Case 1). **(A)** Wound bed preparation (conservative sharp debridement until granulation tissue exceeded 90%). **(B)** Represents the initial application of PRF after wound bed preparation (the first post‑application assessment was performed at week 2). **(C)** After 2 weeks of PRF application, wound area reduction with visible partial epithelialization was observed (the assessment following 2 PRF applications was performed at week 3). **(D)** After 4 weeks of PRF application, marked wound area reduction with increased epithelialization was observed, and the number of PRF applications decreased. **(E)** Wound status after PRF application at week 6. **(F)** At week 7, the wound was almost completely healed (after a total of 6 PRF applications).

#### 3.9.2 Case 2

A 62-year-old man with a history of deep vein thrombosis, varicose veins, marked limb swelling, and prior local skin grafting presented with an active venous ulcer located on the lateral aspect of the left ankle. The ulcer developed after trauma and was aggravated by self-application of anti-inflammatory powder and herbal remedies, leading to local infection and wound enlargement. The ulcer had been present for more than 3 months. The patient declined further hospitalization for skin grafting and preferred conservative treatment.

At initial evaluation, the wound bed consisted of >90% fibrotic slough, with dimensions of 3.5 cm × 7.0 cm (area, 24.5 cm^2^). Conservative sharp debridement was performed to remove non-viable tissue until granulation tissue exceeded 90%. After 8 weeks of wound bed preparation, the patient provided informed consent for PRF therapy, which was provided free of charge. At the start of PRF treatment, the wound area was 3.0 cm × 5.0 cm (area, 15.0 cm^2^). After 2 weeks of weekly PRF applications, the area decreased to 2.0 cm × 4.0 cm (area, 8.0 cm^2^; 46.7% reduction). After 4 weeks, the area was 0.5 cm × 1.5 cm (area, 0.75 cm^2^; 95.0% reduction). A total of 5 PRF applications (one per week) were administered until complete wound closure was achieved. Representative clinical photographs are shown in Figure 6.

**Figure 6:**
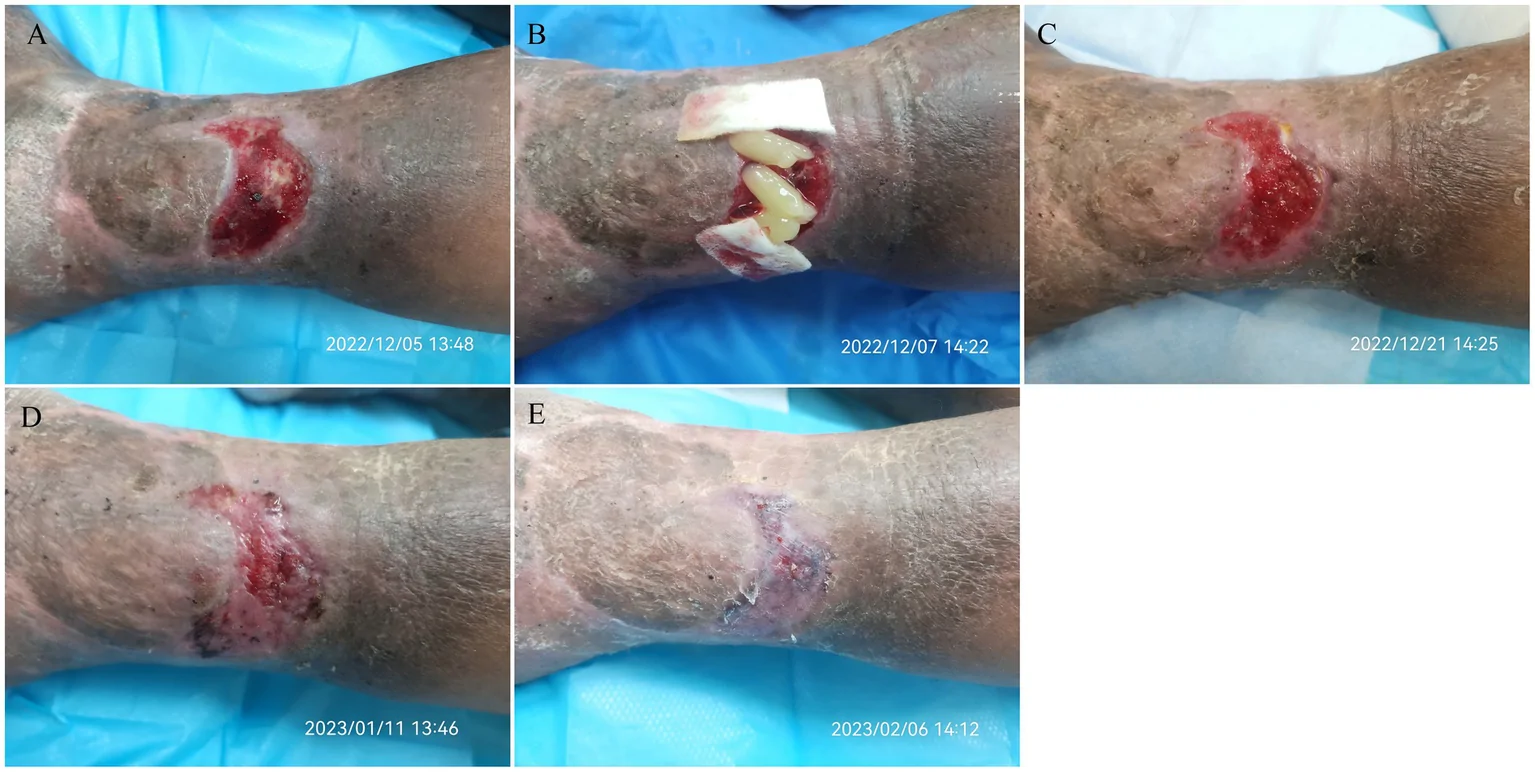
Clinical photographs of a venous leg ulcer treated with PRF-Alginate therapy (Case 2). **(A)** Wound bed preparation. **(B)** Represents the initial application of PRF after wound bed preparation. **(C)** After 2 weeks of PRF application, wound area reduction with visible partial epithelialization was observed. **(D)** After 5 weeks, the wound was nearly covered with epithelialized tissue and was almost completely healed. **(E)** Follow-up photograph.

## 4 Discussion

### 4.1 PRF alginate therapy achieves superior healing and reduces anxiety in active VLUs

This randomized controlled trial demonstrates that autologous platelet-rich fibrin (PRF) gel combined with alginate dressing significantly improves complete wound healing rates in patients with active venous leg ulcers compared to conventional ozonated oil therapy. Notably, this superior healing effect was achieved despite the observation group having significantly larger baseline wound areas and higher venous clinical severity scores, indicating more severe disease at enrollment. Furthermore, patients receiving PRF therapy experienced significantly greater improvements in both state anxiety and trait anxiety scores, indicating that accelerated wound healing translates directly into measurable psychological benefits. As a second-generation platelet concentrate, PRF offers the advantage of providing a three dimensional scaffold rich in platelets, leukocytes, fibrin, and various growth factors (28, 31), key cellular and molecular foundations for wound healing, but its gel form combined with alginate dressing creates a moist, hypoxic “biomimetic” environment, allowing sustained release of bioactive factors that strongly promote fibroblast proliferation, collagen synthesis, and angiogenesis (38). In contrast, ozonated oil, while having certain antibacterial and granulation-promoting effects (12, 39), is far less effective than PRF in providing multiple key growth factors continuously and efficiently (40).

The 83.7% complete healing rate at 12 weeks compares favorably to the 34–80% range documented in recent reviews of advanced VLU therapies (2, 16, 41). Our results are consistent with those reported by O’Connell et al. (17), who also demonstrated the efficacy of platelet-rich fibrin in promoting venous leg ulcer healing. In their prospective pilot study of an autologous platelet-rich fibrin matrix (PRFM) in 17 venous leg ulcers, they reported a complete closure rate of 66.7% and a median healing time of 6 weeks. The mean percentage reduction in the surface area of the ulcers in our study was similar to the results of the other studies (32, 42, 43). The superiority of PRF likely reflects its slow physiological polymerization, which creates a fibrin matrix that entraps high concentrations of growth factors and releases them sustainably over 7 to 14 days (17, 19, 44, 45). This extended interval respects the biological half-life of the PRF fibrin matrix, minimizing mechanical disruption to newly formed granulation tissue and epithelializing edges while maintaining therapeutic growth factor levels throughout the healing cycle. The alginate dressing’s high absorbency and gel-forming properties effectively managed wound exudate over the 7-day period without requiring interim changes, as confirmed by the low rate of periwound maceration observed in the PRF group.

Moreover, this extended interval aligned with PRF’s release kinetics represents a substantial practical improvement over the thrice-weekly changes required in most wound bed preparation protocols (17, 19). This once-weekly regimen thus offers a practical advantage over thrice-weekly changes, particularly for elderly patients with limited mobility and those in geographically remote areas, by reducing clinic visits, transportation burden (46), and overall treatment-related stress.

### 4.2 Patient reported outcomes: anxiety relief and pain control

Chronic wounds impose substantial psychological burdens, including anxiety, depression, pain, and social role loss, particularly among elderly patients with prolonged ulcer duration, large wound area, and recurrent episodes (21, 47, 48). In this study, patients receiving PRF-alginate therapy demonstrated significantly greater improvements in both state and trait anxiety scores. This anxiety-reducing effect is likely mediated through visible healing progress, reduced dressing change frequency (weekly vs. thrice-weekly), and improved wound appearance (including rapid granulation and diminished exudate), which together alleviate concerns about prognosis, economic burden, and social functioning (5, 9). These findings extend prior qualitative observations by quantifying the psychosocial benefits of effective regenerative therapy in VLU management.

Regarding pain, no statistically significant difference was observed between groups at 12 weeks, despite substantial reductions from baseline in both arms. Several factors may explain this convergence. First, both interventions address VLU-associated pain through distinct yet complementary mechanisms: PRF indirectly reduces nociceptive stimuli by accelerating granulation, reducing exudate, and resolving inflammation via leukocyte-mediated immunomodulation (19, 33); ozonated oil exerts direct antimicrobial effects and promotes a clean wound bed, thereby decreasing periwound irritation (39). Second, the Visual Analog Scale-although widely employed may lack sensitivity to capture the chronic, fluctuating pain phenotype characteristic of VLUs, including variations in pain quality (e.g., burning, throbbing) or temporal patterns (e.g., procedural pain, breakthrough episodes). Third, by week 12, PRF-treated patients had achieved complete epithelialization; pain typically resolves following wound closure irrespective of the therapeutic modality (9). Future studies should incorporate validated chronic pain instruments (e.g., McGill Pain Questionnaire, Brief Pain Inventory) and assess procedural pain during dressing changes to comprehensively differentiate analgesic profiles between treatment strategies.

### 4.3 Safety profile: favorable and pragmatically advantageous

No serious adverse events, including severe allergic reactions, wound infection exacerbation, or ulcer deterioration were observed in either treatment arm. The autologous origin of PRF inherently eliminates the immunogenic and pathogen transmission risks associated with allogeneic or xenogeneic biologics (9, 27). Notably, the extended 7-day dressing change interval in the PRF-alginate group did not increase infection rates compared to thrice-weekly changes in the control group, suggesting that alginate dressings provide effective barrier protection while PRF’s antimicrobial and immunomodulatory properties may confer additional resilience against bacterial colonization (9). This favorable safety profile, combined with procedural simplicity and reduced visit frequency, positions PRF-alginate therapy as a practical and scalable option for outpatient and home-care settings where intensive monitoring is not routinely available (42).

### 4.4 Clinical and translational implications: a pragmatic solution for vulnerable populations

This study carries several important implications for VLU management. First, our results demonstrate that early local intervention with PRF-alginate combination can effectively interrupt the vicious cycle of delayed healing and psychological distress in patients with recalcitrant VLUs. The significant reduction in anxiety scores a patient-reported outcome rarely captured in previous wound healing trials provides quantitative evidence that rapid, visible wound improvement positively influences psychosocial well-being. This finding aligns with qualitative studies by Nanninga et al. (49) and Cui et al. (5), who identified chronic wound-related anxiety as a dominant theme in patient narratives. Second, the extended 7–10 days dressing change interval offers particular advantages for elderly, mobility-limited, and geographically isolated populations precisely the demographic groups that bear the disproportionate burden of VLU disease (5, 21, 22, 50). For these patients, each clinical visit requires substantial time, financial resources, and caregiver support (5, 51); reducing visit frequency from three times weekly to once weekly directly addresses documented barriers to care continuity (19, 42, 52). Our protocol achieved this extended interval without increased periwound complications, confirming that the alginate dressing effectively managed exudate and maintained an optimal moist environment throughout the 7-day treatment cycle. Third, the technical simplicity and cost-effectiveness of this approach warrant emphasis. PRF preparation requires only a standard centrifuge and no specialized kits, exogenous additives, or regulatory approvals enabling immediate implementation in outpatient clinics, community health centers, and even resource-constrained primary care settings. This addresses the critical translational gap identified by Hasiba-Pappas et al. (53), who noted that despite compelling biological rationale, few platelet concentrate studies have prioritized pragmatic implementation in real-world clinical environments.

In summary, this study indicates that PRF gel combined with alginate dressing is an effective and safe therapy for promoting healing of venous leg ulcers. It significantly increases complete healing rates while effectively alleviating patient anxiety, reflecting the advantages of the biopsychosocial medical model and holding important clinical value for broader adoption.

## 5 Limitations

Several limitations should be considered when interpreting our findings. First, this was a single-center trial with a modest sample size. Although adequately powered to detect the observed difference in complete healing rates, the sample precluded subgroup analyses to assess whether treatment efficacy varies by ulcer etiology, duration, or comorbid conditions. Multi-center studies with larger, more diverse populations are needed to confirm generalizability. Second, due to inherent differences in the physical appearance and application frequency between PRF gel and ozonated oil, blinding of participants and treating clinicians was not feasible, which may have introduced performance and measurement bias. We attempted to mitigate this by using blinded outcome assessors to measure wound areas from standardized digital photographs. Third, the 12-week follow-up period was sufficient to capture complete healing in most PRF-treated patients but did not allow assessment of long-term outcomes critical to VLU management, particularly ulcer recurrence, scar quality, and healing durability. Given that recurrence rates approach 50–70% within 1 year of closure, extended follow-up is essential to determine whether PRF therapy confers durable benefits. Fourth, we did not quantify growth factor concentrations in the prepared PRF gels or perform histological analysis of wound bed tissue. Consequently, direct correlations between specific biological mediators and clinical outcomes could not be established, constraining mechanistic interpretation. Finally, while ozonated oil combined with alginate dressing represents an active conservative treatment modality routinely used in wound care practice at our institution and has demonstrated favorable efficacy in prior research, however, it may not reflect the standard of care in all healthcare systems, which could limit the generalizability of our findings.

## 6 Conclusion

This study demonstrates that autologous PRF gel combined with alginate dressing is a safe and effective treatment for active venous leg ulcers, with particular advantages for elderly, mobility-limited, and geographically underserved patients. The regimen promotes rapid wound closure, reduces treatment burden, and eliminates the need for costly infrastructure or specialized biologics. These findings support its adoption in outpatient and primary care settings. Future multicenter studies are needed to validate these results, and implementation research should evaluate its cost-effectiveness in vulnerable populations.

## Data Availability

The original contributions presented in the study are included in the article/Supplementary material, further inquiries can be directed to the corresponding author.

## Footnotes

1
www.zstats.net
