# Supplementary material for: Effect of platelet-rich fibrin gel combined with alginate dressing on healing of active venous leg ulcers: a randomized clinical trial
Source: Front Med (Lausanne). 2026 Aug 26;13:1898776. doi: 10.3389/fmed.2026.1898776 (PMC13553255; doi:10.3389/fmed.2026.1898776)
Supplement: Supplementary file 1 [file Supplementary_file_1.docx]

### eTable 1. Stratification of wound area reduction at premature discontinuation of hospital visits

| **Group** | **Wound Area Reduction at Last Visit** | **No. of Patients** | **Time to Discontinuation, weeks (range)** | **Primary Reasons for Discontinuation** |
| --- | --- | --- | --- | --- |
| **PRF-alginate** (n=43) | >85% | 2 | 5-10 | •Transportation difficulties  •Lack of family accompaniment  •Substantial wound improvement, opting for local care |
|  | >75% - ≤85% | 2 | 5-6 |  |
|  | 50% - ≤75% | 3 | 2-3 |  |
|  | Total | **7 (16.3%)** | / |  |
| **Control** (n=43) | >80% - ≤95% | 10^a^ | 6-11 | •Transportation difficulties  •Lack of family accompaniment  •Substantial wound improvement, opting for local care |
|  | >75% - ≤80% | 4 | 4-6 |  |
|  | 50% - ≤75% | 10 | 4-8 |  |
|  | 25%- ≤50% | 4^a^ | 4 | Protocol violation |
|  | >80% | 2^b^ | 12 | / |
|  | Total | **28 (65.1%)** |  |  |

a These 4 patients were switched to PRF treatment because of poor response but remained in the control group for intention-to-treat analysis; data at last assessment were carried forward.
b These 2 patients completed the full 12-week treatment per protocol and achieved >80% area reduction, but did not achieve complete healing; they are not considered “premature discontinuations” but are listed for transparency.
