# Supplementary material for: Effect of platelet-rich fibrin gel combined with alginate dressing on healing of active venous leg ulcers: a randomized clinical trial
Source: Front Med (Lausanne). 2026 Aug 26;13:1898776. doi: 10.3389/fmed.2026.1898776 (PMC13553255; doi:10.3389/fmed.2026.1898776)
Supplement: Supplementary file 2 [file Supplementary_file_2.docx]

**eFigure 1.** An image of the alginate dressing combined with platelet-rich fibrin (PRF) gel.

*Photograph showing the layered structure of the PRF‑alginate dressing: (A) PRF gel after centrifugation and separation. (B) Alginate dressing trimmed to fit wound dimensions, with PRF gel placed onto the trimmed dressing, pre-moistened with platelet poor plasma. (C) Wound bed after debridement and preparation. (D, E) Assembled PRF‑alginate dressing applied to the wound bed, ready for final coverage. The dressing was prepared fresh at each treatment visit and applied within 30 minutes.*
